# Light Stimulation of Neurons on Organic Photocapacitors Induces Action Potentials with Millisecond Precision

**DOI:** 10.1002/admt.202101159

**Published:** 2022-03-18

**Authors:** Tony Schmidt, Marie Jakešová, Vedran Đerek, Karin Kornmueller, Oleksandra Tiapko, Helmut Bischof, Sandra Burgstaller, Linda Waldherr, Marta Nowakowska, Christian Baumgartner, Muammer Üçal, Gerd Leitinger, Susanne Scheruebel, Silke Patz, Roland Malli, Eric Daniel Głowacki, Theresa Rienmüller, Rainer Schindl

**Affiliations:** ^1^ Gottfried Schatz Research Center Chair of Biophysics Medical University of Graz Neue Stiftingtalstraße 6 Graz 8010 Austria; ^2^ BioTechMed‐Graz Graz 8010 Austria; ^3^ Bioelectronics Materials and Devices Laboratory Central European Institute of Technology Brno University of Technology Purkyňova 123 Brno 61200 Czech Republic; ^4^ Department of Physics Faculty of Science University of Zagreb Bijenička c. 32 Zagreb 10000 Croatia; ^5^ Gottfried Schatz Research Center Molecular Biology and Biochemistry Medical University of Graz Neue Stiftingtalstraße 6/6 Graz 8010 Austria; ^6^ Department of Pharmacology Toxicology and Clinical Pharmacy Institute of Pharmacy University of Tuebingen Auf der Morgenstelle 8 72076 Tuebingen Germany; ^7^ NMI Natural and Medical Sciences Institute at the University of Tuebingen 72770 Reutlingen Germany; ^8^ Research Unit of Experimental Neurotraumatology Department of Neurosurgery Medical University Graz Auenbruggerplatz 2.2 Graz 8036 Austria; ^9^ Institute of Health Care Engineering with European Testing Center of Medical Devices Graz University of Technology Graz 8010 Austria; ^10^ Gottfried Schatz Research Center Division of Cell Biology Histology and Embryology Medical University of Graz Neue Stiftingtalstraße 6 Graz 8010 Austria

**Keywords:** bioelectronics, light stimulation, neuronal excitation, OEPC device, photocapacitor, voltage‐gated ion channels

## Abstract

Nongenetic optical control of neurons is a powerful technique to study and manipulate the function of the nervous system. This research has benchmarked the performance of organic electrolytic photocapacitor (OEPC) optoelectronic stimulators at the level of single mammalian cells: human embryonic kidney (HEK) cells with heterologously expressed voltage‐gated K^+^ channels and hippocampal primary neurons. OEPCs act as extracellular stimulation electrodes driven by deep red light. The electrophysiological recordings show that millisecond light stimulation of OEPC shifts conductance‐voltage plots of voltage‐gated K^+^ channels by **≈**30 mV. Models are described both for understanding the experimental findings at the level of K^+^ channel kinetics in HEK cells, as well as elucidating interpretation of membrane electrophysiology obtained during stimulation with an electrically floating extracellular photoelectrode. A time‐dependent increase in voltage‐gated channel conductivity in response to OEPC stimulation is demonstrated. These findings are then carried on to cultured primary hippocampal neurons. It is found that millisecond time‐scale optical stimuli trigger repetitive action potentials in these neurons. The findings demonstrate that OEPC devices enable the manipulation of neuronal signaling activities with millisecond precision. OEPCs can therefore be integrated into novel in vitro electrophysiology protocols, and the findings can inspire in vivo applications.

## Graphical abstract

Using a photocapacitor made from organic pigments, Tony Schmidt and co‐workers successfully stimulate neuronal firing with light. These biocompatible devices convert visible red light into a safe capacitive stimulation for the precise and reliable generation of action potentials in single mammalian cells. This technology enables a wide variety of wireless experimental approaches as well as in vivo applications.

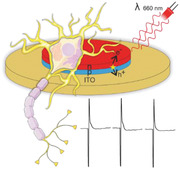

## 1 Introduction

Neural implants constitute powerful tools to therapeutically overcome dysfunctionalities in neural circuits. Therapeutic neuromodulation using electrical stimulation has been well established in clinical use in the past decades. Deep brain and spinal cord stimulation, for instance, provide excellent results in the symptomatic treatment of Parkinson's disease^[^ ^1^, ^2^ ^]^ and in pain treatment, respectively.^[^ ^3^, ^4^ ^]^ In addition, retinal^[^ ^5^, ^6^, ^7^ ^]^ and cochlear implants^[^ ^8^, ^9^, ^10^ ^]^ are successfully used to convert electrical inputs into sensory responses. Engineering of ultrathin, wireless, and organic electronic prostheses represents a promising approach to achieve minimally invasive neuromodulation. The use of light stimulation, in particular, has the potential to eliminate the requirement of wiring neural implants. While the tissue transparency window^[^ ^11^ ^]^ has been an important issue in that quest, high‐performance materials engineered to successfully utilize limited light intensities at the red to near‐infrared allow safe penetration through the skin, connective tissue, or bone.^[^ ^11^ ^]^ Silicon technology can convert light inputs into electric signals to stimulate neuronal tissue.^[^ ^12^ ^]^ Polymers and soft organic electronics possess the mechanical flexibility for use in soft biological tissues, compared to the rigid silicon preparations.^[^ ^13^ ^]^

We have previously developed organic electrolytic photocapacitors (OEPCs) made of semiconductors for the stimulation of cells and tissues.^[^ ^14^, ^15^ ^]^ OEPCs are photovoltaic devices that convert impulses of light into electrochemical displacement currents. The OEPCs are formed of a semiconductor PN junction of metal‐free phthalocyanine (H_2_Pc; p‐type) and *N,N′*‐dimethylperylene‐3,4:9,10‐tetracarboxylic diimide (PTCDI; n‐type). Indium tin oxide on glass serves as a transparent back electrode. The capacitive coupling of the OEPCs with the studied cells was further improved by addition of a thin layer of the conducting polymer formulation, poly(3,4‐ethylenedioxythiophene):poly(styrene sulfonate), PEDOT:PSS to the entire device surface, or only to the back electrode^[^ ^16^ ^]^ in order to increase the electrochemical capacitance and decrease the interfacial impedance.

Upon illumination in a physiological solution, the OEPC is charged and generates a transient electric field to stimulate electrophysiological cell signals in frog oocytes with high temporal and spatial precision.^[^ ^15^ ^]^ Traditionally, photocapacitive stimulation is considered a safer mechanism in comparison to faradaic or photo‐thermal stimulation that could result in cell or tissue damage due to reactive oxygen species or heat generation.^[^ ^17^ ^]^ OEPCs absorb light efficiently due to the high absorbance coefficient of the organic semiconductors, allowing these devices to be tens of nanometers thick. OEPCs produce effectively transductive extracellular potentials in a physiological solution upon illumination using light within the tissue transparency window at 630 to 660 nm wavelength. We have previously established electrophysiological ion channel recordings in single *Xenopus laevis* oocytes, finding rapid, micro‐ to millisecond photoinduced transient changes in the range of 20 to 110 mV.^[^ ^15^, ^16^ ^]^ All this previous work on OEPCs has validated their efficacy in stimulating larger targets of oocytes, retinal tissue explants, and peripheral nerves.^[^ ^18^ ^]^

The aim of this work is to establish OEPCs for single cells for in vitro electrophysiology experiments, in order to both better understand the operation principles, but also to introduce this technology as a tool for biophysicists. The size of a single neuron is ≈100‐fold smaller than the previously investigated *Xenopus* oocytes and it possesses a repertoire of fine‐tuned and fast responding voltage‐gated ion channels, which open upon membrane depolarization. On a molecular basis, voltage‐gated ion channels contain a positively charged voltage sensor that moves upon depolarization. This voltage‐sensor transition is directly coupled to open the pore of the voltage channels.^[^ ^19^ ^]^ As the plasma membrane is extremely thin, the membrane potential generates an electric field of ≈10^7^ V m^–1^ to induce conformational changes in voltage‐gated channels.^[^ ^20^ ^]^ The OEPC is in principle an extracellular stimulation electrode, with a cathodic leading phase. This is because upon illumination, the n‐layer of OEPC charges negatively. Cells in close contact with the OEPC surface experience an extracellular electric field. The extracellular potential change mainly polarizes the adhered plasma‐membrane fraction.^[^ ^15^, ^21^ ^]^ Contrary to this attached membrane, the electric field of OEPC leads to a hyperpolarization of the free membrane at the same time.^[^ ^15^, ^21^ ^]^ A limiting stimulatory parameter is the geometry of the semiconductor–cell interface. In our work here, the attached cell membrane is separated from the OEPC surface through the glycosylation layer and the extracellular medium. The size of the electrolyte filled cleft between the OEPC and the attached cell membrane is critical for the modulation of the membrane potential, since the electrical potential exponentially decays with distance. Similarly, the surface area of the OEPC relative to the cell's attached membrane area is an important factor for the efficacy of the stimulation. OEPCs of a similar size to the stimulated cell may more efficiently couple the generated photocurrents to the cell due to the details of the geometrical electric field distribution, however they may not be able to generate sufficient current density to efficiently drive the neuromodulation. Moreover, the number of depolarizing ion channels in the attached cell membrane controls the size of generated ion currents and membrane polarization.^[^ ^22^ ^]^

In this study, we first determined the efficiency of OEPCs on the activation of voltage‐gated ion channels in HEK293 cells with heterologous expression of the potassium channel K_v_1.3, a member of the shaker‐related subfamily. For electrophysiological patch‐clamp recordings and subsequent electrical circuit simulations, the currents of slowly inactivating potassium channel K_v_1.3 voltage‐gated ion channel were used to recalculate the time‐dependent membrane polarization induced by OEPC mediated stimulation. We defined an OEPC stimulation model including a hidden Markov model (HMM) representing the kinetics of K_v_1.3 ion channels to compare the effect of external stimulation through OEPCs to internal stimulation of the cell in voltage‐clamp measurements. Based on these simulations, we can show the increase in ion channel conductivity in response to OEPC stimulation. The model is further used to simulate the effect of external OEPC stimulation in voltage‐ and current‐clamp mode separating the effect on the attached and free parts of the membrane.

We here show that millisecond light pulse protocols could efficiently activate heterologously expressed voltage‐gated ion channels and trigger neuronal action potentials (AP) firing in single recorded cells of neuronal cultures. Our electrophysiological recordings and electrical models determine that OEPC mediated AP firing is generated by depolarization of the attached membrane.

## 2 Experimental Section

### 2.1 Cell Culture and Cell Viability Test

HEK293 cells stably expressing TagRFP‐K_v_1.3 used for patch‐clamp experiments were generated as previously described.^[^ ^23^ ^]^ In short, cells were transfected with a linearized plasmid, containing the TagRFP‐K_v_1.3 sequence, cultured with G418 (Genetecin) and sorted by FACS (FACS Aria IIIu, BD Biosciences, Heidelberg, Germany) analysis. For cell viability tests, 75.000 HEK293 cells were seeded on OEPCs and cultivated in 24‐well plates with 1 mL Dulbecco's Modified Eagle's medium (DMEM) supplemented with 10% fetal bovine serum (FBS). Control cells were seeded on glass coverslips. Cell viability was assessed using a colorimetric MTS assay (CellTiter 96 AQueous One Solution Cell Proliferation Assay, Promega, Germany) after 24, 48, 72, and 96 h according to manufacturer's protocol and the luminance units were measured using a CLARIOstar platereader (BMG Labtech, Germany) at 490 nm wavelength after 1 h incubation with the assay reagent. Cell viability was normalized to control conditions for each time point. Hippocampal neurons of postnatal Sprague Dawley rats (P0‐1) (Charles River) were prepared as previously described.^[^ ^24^ ^]^ On a single 30 mm OEPC device, an average of 170.000 cells were seeded containing neuronal and glial cells. For cell viability tests, the homogenate of the hippocampi from 4 pups was seeded onto 96‐well plates with or without an OEPC surface coated with poly‐d‐lysine in 150 µL neuronal medium.^[^ ^24^ ^]^ Cell viability was assessed every 72 h over a course of 15 d using MTS assay as described above. The same preparation of primary neurons was used on 30 mm OEPC devices in patch clamp experiments. Cell viability was not significantly altered in both conditions compared to their control groups (multiple unpaired *t*‐test analysis, desired FDR = 1.00%).

### 2.2 OEPC Fabrication

Round 30 mm diameter glasses coated with indium tin oxide (ITO) (Kintec, Hong Kong) were sonicated sequentially in acetone, isopropanol, 2% Hellmanex III solution, and deionized water. Finally, the substrates were treated with oxygen plasma (100 W, 5 min, Diener electronic GmbH). The substrates were then exposed to n‐octyltriethoxysilane (OTS) vapor in a steel Petri dish heated to 90 °C for 1 h. The samples were subsequently sonicated in acetone, and washed with isopropanol and deionized water to remove multilayers of OTS. In some samples, the OTS treatment was patterned by a PVC adhesive tape. Next, the organic semiconductors phthalocyanine H_2_Pc (Alfa Aesar) and *N,N′*‐dimethyl‐3,4,9,10‐perylenetetracarboxylic diimide (PTCDI) (BASF), both purified by threefold temperature‐gradient sublimation, were deposited in a PVD chamber through a PVC adhesive tape mask. The process was done at <2 × 10^−6^ Torr, rate of 1–5 Å s^–1^ and 30 nm of each material were successively evaporated to produce the organic pixel (PN). The thermal control (indigo, BASF) samples were processed similarly, except the evaporated layer was 60 nm thick to offset the higher absorption coefficient of phthalocyanine absorber. Some of the devices were coated with poly(3,4‐ethylenedioxythiophene):poly(styrene sulfonate), or PEDOT:PSS solution. PEDOT:PSS (Clevios PH 1000, Heraeus), dimethyl sulfoxide (1 w%), and 3‐glycidoxypropyltrimethoxysilane (2 w%) were sonicated together for 10 min and drop casted at the return electrode or spin coated (5000 rpm, 30 s) over the PN photopixel. The PEDOT:PSS devices were then annealed at 140 °C for 90 min. Finally, all devices were tested for functionality with the electrophotoresponse technique as previously described.^[^ ^15^ ^]^

### 2.3 Scanning Electron Microscopy (SEM)

Primary neuron culture and HEK cells were grown on the OEPC devices and fixed in 2% paraformaldehyde and 2.5% glutaraldehyde in 0.1 m cacodylate buffer pH 7.4 for 30 min. After washing for 30 min in 0.1 m cacodylate buffer pH 7.4 the samples were dehydrated in a graded series of ethanol of 30%, 50%, 70%, 80%, 90%, and 96% for 15 min each, followed by 2 × 5 min in 100% (p.a.) ethanol and transferred to 100% (p.a.) acetone. The samples were dried using a BalTec CPD 030 critical point dryer (Balzers, Liechtenstein) and sputter coated using a BalTec SCD 500 sputter coater (Balzers, Liechtenstein). Imaging was performed with a Zeiss Sigma 500 VP scanning electron microscope (Zeiss, Oberkochen, Germany) operated at 2 kV, using an Everhart‐Thornley secondary and backscattered electron detector. Images were acquired with the Zeiss SmartSEM imaging software.

### 2.4 Electrophysiology

For patch‐clamp recordings of stably expressed K_v_1.3 channels in HEK293 cells, the intracellular solution contained 145 × 10^–3^ m KCl, 1 × 10^–3^ m MgCl_2_, 10 × 10^–3^ m HEPES (2‐[4‐(2‐Hydroxyethyl)piperazin‐1‐yl]ethane‐1‐sulfonic acid), and 10 × 10^–3^ m glucose adjusted to pH 7.4 with KOH. The bath solution contained 140 × 10^–3^ m NaCl, 5 × 10^–3^ m KCl, 1 × 10^–3^ m MgCl_2_, 10 × 10^–3^ m HEPES, and 10 × 10^–3^ m glucose adjusted to pH 7.4 with NaOH. Voltage clamp step protocols were clamped at a holding potential of ‐100 mV for 2.5 s followed by a depolarization step of 400 ms ranging from ‐100 to 40 mV in 10 mV steps. After 20 ms of depolarization a 5 ms light pulse was applied with a 10 W LED emitting at 660 nm (Roschwege, Germany) mounted to the objective revolver for bottom illumination of the OEPC device. For neuron experiments, the perforated patch method was used, offering the advantage of long‐term stable patch‐clamp seals together with near normal cytoplasm composition. However, resting membrane potentials might be slightly depolarized. Pipette solution contained 3.5 × 10^–3^ m NaCl, 1.5 × 10^–3^ m CaCl_2_, 0.25 × 10^–3^ m MgCl_2_, 10 × 10^–3^ m HEPES, 120 × 10^–3^ m d‐gluconic acid potassium salt, 1.5 × 10^–3^ m d‐gluconic acid sodium salt, 5 × 10^–3^ m EGTA (3,12‐bis(carboxymethyl)‐6,9‐dioxa‐3,12‐diazatetradecane‐1,14‐dioic acid) adjusted to pH 7.3 with KOH; shortly before the experiment we added 250 µg mL^–1^ amphotericin B dissolved in dimethyl sulfoxide (DMSO). The bath solution contained 140 × 10^–3^ m NaCl, 2.5 × 10^–3^ m CaCl_2_, 2 × 10^–3^ m MgCl_2_, 2 × 10^–3^ m KOH, 10 × 10^–3^ m HEPES, and 10 × 10^–3^ m glucose adjusted to pH 7.4 with NaOH. For voltage‐clamp protocols neurons were clamped at ‐70 mV for 3 s followed by a depolarization step of 500 ms ranging from ‐70 to 20 mV in 5 mV steps. After 20 ms a light pulse with varying length and intensity was applied with a 700 mW diode laser emitting at 638 nm (Lasertack, Germany). Reversal potential for K_v_1.3 was recorded from tail currents (Figure S2C, Supporting Information). Neurostimulation in current‐clamp mode was performed with *I* = 0 pA. Whole‐cell recordings were performed at room temperature with an insulating bath chamber made from polytetrafluoroethylene and an Ag/AgCl electrode as reference. Experiments with Tetrodotoxin (TTX) were performed with a gravity‐based perfusion system and a perfusion chamber (NGFI, Graz, Austria) filled with the neuronal bath solution containing no or an additional amount of 0.5 × 10^–6^ m TTX. First‐order derivatives (d*V*/d*t*) of AP slopes are derived from traces filtered with a lowpass Bessel 8‐pole filter at 5 kHz.

### 2.5 Confocal Microscopy, 3D Convolution, and Membrane Area Calculation

HEK293 cells were transfected for 24 h with mTagRFP‐Membrane‐1 that was a gift from Michael Davidson (Addgene plasmid # 57992). 3 h before imaging, cells were checked for an even membrane fluorescence and reseeded to obtain single cells attaching to the surface. Z‐stacks of 12‐bit images were taken at a Nikon A1R confocal microscope (Japan) using a Nikon Plan Apo 40×/0.95 objective, with image size of 1024 × 1024 pixels, pixel dwell time of 1.1 µs, pixel size = 0,31 µm per px, Z‐step = 0.374 µm and four times averaging. The Amira software 6.5.0 (Thermo Fisher Scientific) was used for 3D convolution, mesh generation, and surface area calculation.

### 2.6 Mathematical Modeling and Simulation

Parameter estimation and simulation were done using MATLAB/Simulink 2019a (Mathworks). To quantify the effect of OEPC mediated stimulation, we defined a model for the whole‐cell patch clamp recordings of heterologously expressed TagRFP‐K_v_1.3 channels in HEK293 cells.^[^ ^25^ ^]^ The total cell membrane current model *I*_mem_ is composed of a background current *I*_back_, a capacitive current caused by the capacitive nature of the cell membrane and the modeled *I*_Kv1.3,TagRFP_ and can be described using the following equation:

(1) $$\begin{array}{c} I_{\text{mem}}=C_{M}\frac{dV}{dt}+I_{\text{Kv}1.3,\text{TagRFP}}\left(t,V\right)+\;I_{\text{back}} \end{array}$$

where *V* is the membrane voltage, *C*_M_ is the total capacitance of the cell membrane, and *t* is the time. Our kinetic model of *I*_Kv1.3,TagRFP_ simultaneously simulates the whole kinetics of the TagRFP‐K_v_1.3 channel including activation, deactivation, and recovery. Single‐channel kinetics were modeled by a hidden Markov model. We used an additional independent variable *N* to fit the macroscopic currents to the recorded whole‐cell currents.^[^ ^26^ ^]^ Thus, *N* represents the number of active channels in a specific cell. The parameters for the *I*_Kv1.3,TagRFP_‐model were estimated using global constrained optimization (Particle Swarm Optimization PSO, particleswarm MATLAB Mathworks) based on different activation and deactivation protocols in whole‐cell patch‐clamp experiments (Figure S2A–C, Supporting Information). The starting value of the intrinsic cell parameters (membrane resistance *R*_B_,*C*_M_) and patch parameters (series resistance *R*_S_,seal resistance *R*_seal_) is obtained based on the built‐in functionality of the patch‐clamp system and further optimized to fit the patch‐clamp measurements (Particle Swarm Optimization PSO, particleswarm MATLAB Mathworks) based on the activation protocol steps. We assume that the amplifier provides the clamp voltage *V*_c_ as specified and the cell parameters and patch parameters *R*_B_,*R*_S_, *C*_M_ and *R*_seal_ remain constant over the course of one sweep. One does not model any voltage dependence of *C*_M_. The filter was defined as a fourth order Bessel filter with cutoff frequency ω_0_ = 2π 2000 Hz (MATLAB besself and impinvar function for discretization) and applied using the MATLAB filter function. The model is used to reproduce the ion currents through the cell membrane and capacitive transients upon voltage stimulation and serves as a basis for the OEPC stimulation model.

### 2.7 Kinetic Model for *I*_Kv1.3,TagRFP_

The kinetics of the voltage and time‐dependent potassium current *I*_Kv1.3,TagRFP_ are modeled as a first‐order hidden Markov model (HMM) which represents the gating of an ion channel through a series of conformational changes (states) of the channel protein, where the transition probability between these states depends on the present state only. The state transition probabilities are either voltage dependent and given in the form: $\alpha \;=\alpha_{1}\;\cdot \;{\text{exp}}^{\left(\frac{\alpha_{2}\cdot V\cdot F}{R\cdot T}\right)}$ for the forward and $\beta \;=\beta_{1}\;\cdot \;{\text{exp}}^{-\left(\frac{\beta_{2}\cdot V\cdot F}{R\cdot T}\right)}$ for the reverse transitions, where α_*i*_ and β_*i*_ represent specific gating parameters, *V* the voltage across the cell membrane, *F* the Faraday constant, *R* the gas constant and *T* the temperature or non‐voltage‐dependent transition probabilities *A* and *B*. Defining $P_{S_{i}}(t)$ as the probability of being in a specific state *S_i_* at time *t* leads to the equation for the time evolution of the channels’ probability of being in the open state *P*_O_(*t*):^[^ ^27^ ^]^

(2) $$\begin{array}{c} \begin{array}{l} \frac{dP_{O}}{dt}=\underset{j}{\sum }P_{S_{j}}\;\cdot \;\mathrm{incoming}\;\text{transition}\;\text{from}\;S_{j}\;\\ -\underset{j}{\sum }P_{O}\;\cdot \;\text{outgoing}\;\text{transition}\;\text{to}\;S_{j},\;j\text{…number}\;\text{of}\;\text{states} \end{array} \end{array}$$

For sufficiently large numbers of the same channel, the quantities in this equation can be replaced by their macroscopic interpretation and the probability of being in a state *S_i_* can be interpreted as the fraction of channels in state *S_i_* . The ion current can thus be defined as

(3) $$\begin{array}{c} \begin{array}{l} I_{\text{Kv}1.3,\text{TagRFP}}\left(t,V\right)=\;N\;\cdot \;g_{\text{Kv}1.3,\text{TagRFP}}\cdot P_{o}\left(t\right)\;\cdot \;\left(V-E_{\text{Kv}1.3,\text{TagRFP}}\right)\\ =G_{\text{Kv}1.3,\text{TagRFP}}\;\cdot \;\left(V-E_{\text{Kv}1.3,\text{TagRFP}}\right) \end{array} \end{array}$$

where *N* is the estimated number of TagRFP‐K_v_1.3 channels, *g*_Kv1.3,TagRFP_ the single channel conductance and *E*_Kv1.3,TagRFP_ the reversal potential. *P*_o_(*t*) describes the probability for a single channel of being in the open (conducting) state and comes from the TagRFP‐K_v_1.3 model.^[^ ^26^ ^]^ $G_{M}=G_{\text{Kv}1.3,\text{TagRFP}}=\frac{1}{R_{M}}\;=\;N\;\cdot \;g_{\text{Kv}1.3,\text{TagRFP}}\;\cdot \;P_{o}(t)$ defines the conductance of all TagRFP‐K_v_1.3 channels (marked with an arrow in Figure 2A). The parameters were derived from experiments performed in TagRFP‐K_v_1.3 transfected HEK293 cells to model the activation and deactivation, respectively (Figure S2A–C, Supporting Information) using particle swarm optimization with a hybrid function (particleswarm MATLAB Mathworks, global optimization toolbox, swarm size, 200, hybrid function fmincon).^[^ ^28^ ^]^ The model bounds were based on the description of Kv1.3 in ref. [26] and set to lower bound = [100, 0.01, 90, 0.1, 2000, 1500, 8000], upper bound = [500, 0.5200, 1, 50 000, 9000, 20 000] for the set of parameters $\left[\alpha_{1},\beta_{1},m\;=\frac{R\;\cdot \;T}{\alpha_{2}\;\cdot \;F}\;,n\;=\frac{R\;\cdot \;T}{\beta_{2}\;\cdot \;F}\;,A,B,N\right]$, respectively. The parameter *N* was further adapted for each individual cell to fit the measurements using the MATLAB fminbnd function to minimize the quadratic relative difference between the steady‐state current simulations and measurements.

### 2.8 Equivalent Circuit Model for the *I*_TagRFP‐Kv1.3_ Ion Current

The entire equivalent circuit (Figure 2A) including cell‐specific parameters and patch‐clamp parameters (*C*_M_,*R*_B_,*R*_S_,*R*_seal_), and the *I*_TagRFP − Kv1.3_ ion current model was implemented in MATLAB/Simulink as Linear Parameter varying (LPV) Model with the clamp voltage provided by the patch‐clamp amplifier as input *V*_C_ and the current measured by the patch clamp system as output *I*_out_. The model allows to quantify the membrane voltage of the cell membrane, which may differ from the provided clamp voltage *V*_C_′ due to the access resistance *R*_S_. The patch clamp amplifier and the resistance and capacitance compensating circuits are not designed for measuring the effects of external stimulation. Thus, the access resistance was not compensated during the measurements but instead included in the model. Detailed model equations for the *I*_TagRFP − Kv1.3_ ion current model can be found in the Supporting Information (model equations).

Biocompatible surfaces, such as the OEPC surface, have the property that living cells can adhere to them. The adhesion is limited to a part of the membrane of the cell, which is then termed the attached part of the membrane.^[^ ^21^ ^]^ The very small gap between the attached membrane and the biocompatible surface is essentially filled with the bath electrolyte. This is also true for any biocompatible surface, as for example the surface of the Petri dish. To comply with the following OEPC stimulation model, the membrane is split into an attached and a free part.

### 2.9 OEPC Stimulation Model

To quantify the effect of OEPC stimulation on the cell membrane of the entire cell, the previously defined two domain model for the extracellular stimulation using electrolyte–oxide–semiconductor capacitors was adapted.^[^ ^21^, ^29^ ^]^ In this model, the cell membrane with a total surface area of *A*_M_ is split in two parts. One part is attached to the OEPC device with a total area *A_J_* . This attached part of the cell membrane causes a high‐ohmic seal between the cleft underneath the cell and the electrolytic bath. The remaining part of the cell membrane with a surface area of *A*_M_ − *A*_J_ is called the free membrane. The voltage across the free membrane, or more precisely, the voltage drop from the pipette to the external reference electrode, is mainly controlled by the patch clamp system in voltage‐clamp mode, i.e., the feedback loop will try and keep *V*_C_′ (voltage at the inverting input of the amplifier) and *V*_c_ at the same level, whereas the attached membrane is defined by the voltage *V_I_* − *V_J_*, where *V_J_* describes the voltage in close proximity to the attached cell membrane on top of the OEPC device and *V_I_* the potential in the inside of the cell. The total membrane current consists of the sum of the current caused by the attached and the free membrane (Figure 2A):

(4) $$\begin{array}{c} i_{\text{mem}}\;=i_{\text{attached}}+i_{\text{free}} \end{array}$$

Both parts of the membrane provide the model current similar to Equation (1):

(5) $$\begin{array}{c} \begin{array}{l} i_{\text{free}}=A_{MJ}\;\cdot \;\left(G_{B}+G_{M,\text{free}}\right)\;\cdot \;V_{M,\text{free}}-A_{\text{MJ}}\;\cdot \;G_{B}\;\cdot \;E_{\text{VHEK}}\\ -A_{\text{MJ}}\;\cdot \;G_{M,\text{free}}\;\cdot \;E_{VK}+C_{M}\;\cdot \;A_{\text{MJ}}\;\cdot \;\frac{dV_{M,\text{free}}}{dt} \end{array} \end{array}$$

(6) $$\begin{array}{c} \begin{array}{l} i_{\text{attached}}=A_{J}\;\cdot \;\left(G_{B}+G_{M,\text{attached}}\right)\;\cdot \;V_{M,\text{attached}}-A_{J}\;\cdot \;G_{B}\;\cdot \;E_{\text{VHEK}}\\ -A_{J}\;\cdot \;G_{M,\text{free}}\;\cdot \;E_{VK}+C_{M}\;\cdot \;A_{\text{MJ}}\;\cdot \;\frac{dV_{M,\text{attached}}}{dt} \end{array} \end{array}$$

where the conductivities *G*_M,free_ and *G*_M,attached_ are given by the K_v_1.3‐model, *V*_M,free_ is the voltage across the free membrane *V*_I_ − *V*_E_, *V*_M,attached_ is the voltage across the attached membrane *V*_I_ − *V*_J_, *A*_MJ_ and *A*_J_ define the size of the free and the attached membrane, respectively. The resistance *R*_J_ defines the seal between the cell and the adhesive surface and is connected to the ground via the electrolytic bath.

## 3 Results

### 3.1 Light‐Triggered Activation of Ion Channels

**Figure 1:**
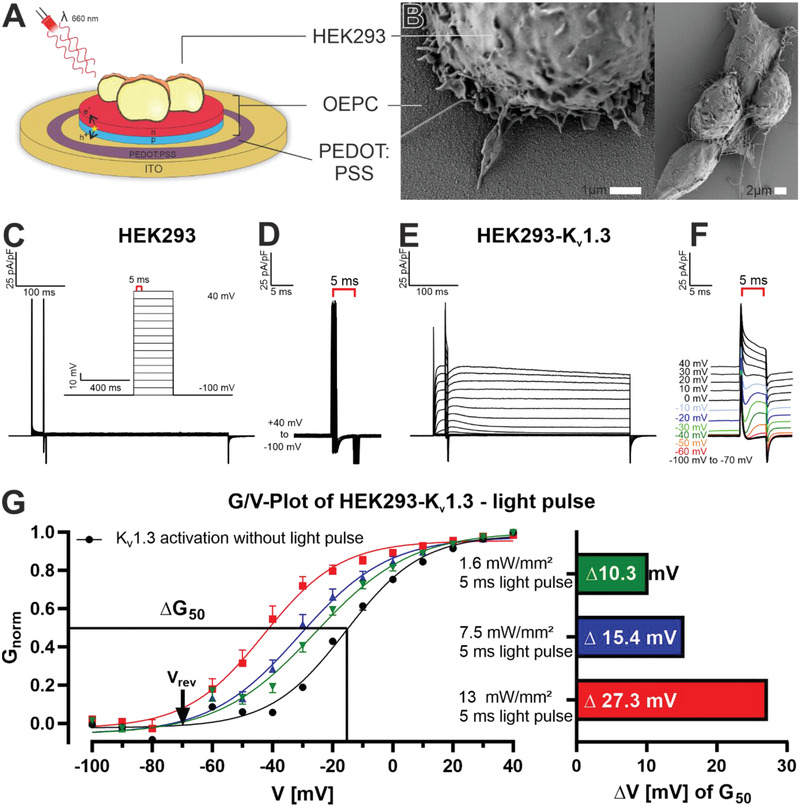
Light regulation of K_v_1.3 channels expressed in HEK293 on OEPCs by patch clamp recordings. A) Schematic representation (not to scale) and B) SEM imaging of HEK293 cells on OEPCs with a drop‐casted PEDOT:PSS ring. C) Step‐protocol of clamped HEK293 on OEPCs D) with zoom into the 5 ms LED pulse of 13 mW mm^–2^ at 660 nm. E) Step‐protocol of clamped HEK293‐K_v_1.3 on OEPCs F) with zoom into the 5 ms LED pulse of 13 mW mm^–2^ at 660 nm. G) Conductance/voltage‐plot are presented as mean values (+SEM) and *G*_50_ at three different light intensities 13 mW mm^–2^ (*n* = 17), 7.5 mW mm^–2^ (*n* = 10), 1.6 mW mm^–2^ (*n* = 9) and no light stimulation (*n* = 17).

We initially investigated the contact of single mammalian cells on OEPC devices. For our first experiments, we used a ring of PEDOT:PSS surrounding the OEPC photo layer (**Figure** **1**A) to further improve capacitive coupling upon light illumination. Already three hours after seeding, HEK293 cells formed filopodia on the crystalline PTCDI surface of the semiconductor (Figure 1B). The cell viability of the cell culture was comparable to the control groups that were cultivated on plain glass coverslips and did not show any significant difference (Figure S1A, Supporting Information).

Voltage‐gated Na^+^, Ca^2+^, and K^+^ channels are the main contributors of ion exchange that constitutes AP firing. Therefore, we further utilized a human HEK293 cell line with a heterologous expression of voltage‐gated K_v_1.3 channels to assess their activity in response to the electric field created by capacitive charging of the OEPC device during temporally controlled illumination protocols. K_v_1.3 belongs to the class of slowly or noninactivating potassium ion‐conducting channels in the shaker‐related subfamily. As a delayed rectifier, K_v_1.3 potassium currents repolarize the membrane potential after AP firing. Two distinct features make the ion channel a perfect candidate for the light‐triggered activation of ion channels: first, this K^+^ channel is activating within milliseconds, which suits the fast photocapacitive coupling process of the OEPC devices. Second, its slow C‐type inactivation allows combining voltage‐clamped subthreshold membrane potentials with light pulses. These experiments aimed not only to determine how single light pulses flashed on OEPC devices stimulate voltage‐gated ion channels in live‐cell experiments but also to recalculate time‐dependent local membrane polarization and channel conductivity in electric circuit simulations.

In the following experiments, we used voltage‐clamp recordings in whole‐cell configuration with HEK293 cells stably expressing TagRFP‐K_v_1.3 channels (named here K_v_1.3 channels) directly grown on OEPCs. Depolarizing voltage steps in 10 mV increments (inset in Figure 1C) were applied via the patch pipette electrode to single cells from a holding potential of ‑100 mV to a maximum voltage of +40 mV. K_v_1.3 current activation was monitored starting from a clamped membrane potential of ‐30 mV that yielded a strong outward current. More positive holding potentials correlated with larger K_v_1.3 currents (Figure 1E and Figure S1B, Supporting Information, black curve). Typically, K_v_1.3 currents reached a steady‐state plateau within 10 ms. A subsequent 5 ms illumination (13 mW mm^–2^) pulse resulted in a positive and negative current transient (a typical capacitive charging/discharging behavior) when switching the light on and off, respectively. Upon sub‐threshold steps between ‐60 and ‐40 mV, no K^+^ currents were determined without light stimulation. Importantly, upon light stimulation (red, orange, and dark green trace, Figure 1F) a typical and robust additional K^+^ efflux was recorded. Specifically, the light pulse induced an additive K_v_1.3 outward current on top of the current induced by clamping the membrane between ‐30 and 0 mV. The time‐course of K_v_1.3 current recordings yielded the same typically delayed activation characteristics of the K_v_1.3 channel, induced by voltage steps and light pulses. At a holding potential of +10 mV, K_v_1.3 channels reached maximum conductance; hence additional light pulses only resulted in current inactivation (Figure 1F). As a control, transfected HEK293 cells on glass coverslips responded to voltage steps but lacked the characteristic K^+^ currents during the light pulse (Figure S1C,D, Supporting Information). In addition, nontransfected HEK293 cells on OEPCs resulted in only capacitive transients when the light was turned on and off (Figure 1C,D).

Analogous experiments with reduced light intensities yielded a graded shift in K^+^ current activation (Figure 1G). We calculated the half‐maximal conductivity Δ*G*_50_ of the K_v_1.3 channels for all conditions for a measured reversal potential of ‐73 mV (Figure S1E, Supporting Information). In the absence of a light stimulus, K_v_1.3 ion channels reach G_50_ at ‐16 mV (Figure 1G, left). Instead, a 5 ms pulse with an intensity of 13 mW mm^–2^ shifts the Δ*G*_50_ of K_v_1.3 by 27 mV. At light intensities of 7.5 mW mm^–2^ and 1.6 mW mm^–2^ Δ*G*_50_ shifts by about 15 and 10 mV, respectively (Figure 1G, right). These experiments show a light intensity‐dependent graded holding potential shift for K^+^ channel activity. For the highest intensity, OEPCs induce a net membrane potential shift of ≈30 mV to activate K^+^ currents already at a membrane potential of ‐60 mV.

### 3.2 Mathematical Modeling of the Ion Channel and Membrane Potential Simulation

**Figure 2:**
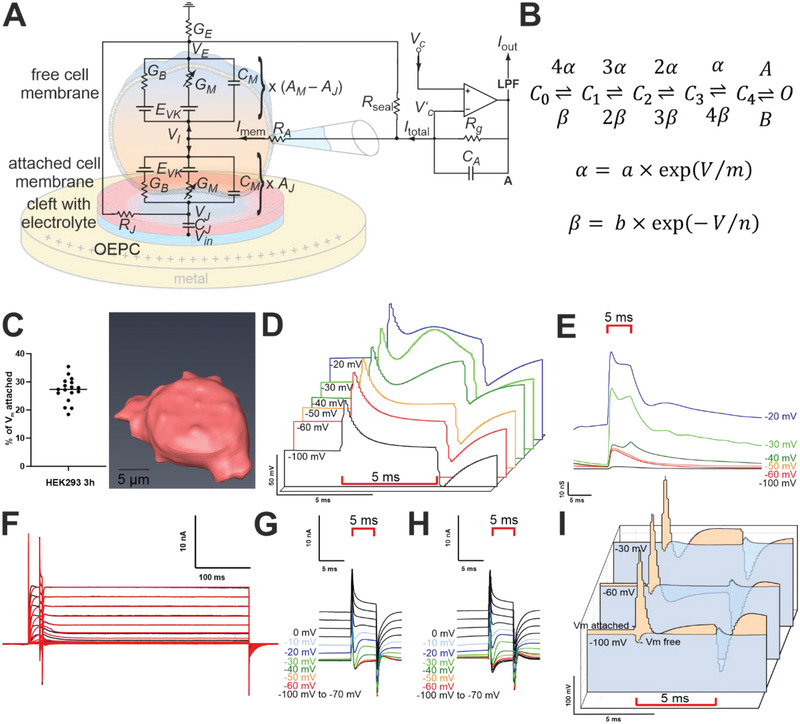
Modeling of K_v_1.3 gating and OEPC induced K_v_1.3 conductance increase and membrane polarization. A) Electric circuit including a two domain model for OEPC stimulation with the membrane split into the attached and free part. B) Open and closed states of TagRFP‐K_v_1.3 and probability parameters. C) 3D convolution of HEK293 cells and percentage of the attached membrane on the whole surface 3 h after seeding (*n* = 17). D) Simulated equivalent voltage‐clamp input *V*_C_′. This clamp‐protocol provokes the same ion current *I*_out_ as the external OEPC stimulation. E) Modeled ion channel conductance of the cell membrane during light illumination. F) HEK measurement (black) and model simulation (red). The voltage step and light illumination protocol are the same as in Figure 1C. G) single HEK293‐K_v_1.3 measurement on OEPC and H) modeled currents. I) Modeled membrane voltage time‐course for the free *V*_M,free_ and the attached *V*_M,attached_ membrane generated due to light pulses and the voltage clamp amplifier set to different command potentials.

Previous experiments in oocytes have shown a time and spatial‐dependent membrane voltage polarization due to external voltage stimulation. The spatial membrane polarization can be grouped into a two‐domain model;^[^ ^21^ ^]^ the attached membrane, *V*_M,attached_, in close vicinity to the OEPC surface and the free membrane *V*_M,free_. Extracellular electrical stimulation resulted in a different polarity of the attached membrane and the free cell membrane.^[^ ^15^ ^]^ For *V*_M,attached_, the coupling between cell and OEPC is determined by the electrical properties of the OEPC surface, the size of the cleft and the cell membrane, which can be derived from the geometry of the adhesion and area‐specific parameters. We set up an electrical circuit to calculate time‐dependent membrane polarization in dependence of the cell geometry (**Figure** **2**A). The voltages *V*_I_ in the cell, *V*_J_ in the cleft and *V*_E_ in the electrolytic bath near the cell are provided related to the potential of the bath electrode, although the OEPC device is electrically floating, and the back electrode (ITO) is not directly connected to the ground. The dynamics are determined by the current balance in the cell, in the gap, and the bath as well as the patch‐clamp amplifier in the voltage‐clamp mode which is trying to keep the tip of the patch electrode at a predefined value. All the quantities are location‐dependent; however, a simplified description of the system is still valuable for showing differences in the attached and free parts of the membrane and the comparably small HEK cells. Thus, the well‐established two domain model for the external stimulation of cells is adapted to the OEPC stimulation. During light stimulation, the membrane attached to the OEPC is depolarized in the beginning, which causes voltage‐gated ion channels to open. On the opposite site, the free membrane is hyperpolarized and does not contribute to ion channel activity in current clamp mode.

In order to determine the relative sizes of the attached and free part, we estimated the surface area of the attached and the free membrane of HEK293 cells on OEPCs by Z‐stack confocal microscopy 3 h after seeding. The cells differ in size but after 3D convolution (Figure 2C and Figure S2D, Supporting Information) their ratio of attached to the free membrane was determined to be the median of 27.36 % (Figure 2C, *n* = 17, interquartile range: 24.7% to 30.0%). This information was used in the model to define the membrane areas *A_J_* and *A_M_* of the free and attached parts, respectively. Upon illumination, high density charging currents drive the transductive extracellular potentials in the cleft between the cell and the OEPC.

To determine the actual membrane voltage caused by light stimulated OEPCs, we generated a sequential Hidden Markov model (Figure 2A) describing the whole kinetics of the over‐expressed K_v_1.3 channels in HEK cells. This detailed ion current model serves as a basis for the conductivities in the OEPC stimulation model and was adapted from the wild type K_v_1.3 model^[^ ^26^ ^]^ which shows a different inactivation. The original model for the wild type K_v_1.3 includes an additional inactive state that was eliminated during optimization. Our channel model includes the forward (activation) rates $\alpha \;=\;\alpha_{1}\;\cdot \;{\text{exp}}^{\left(\frac{\alpha_{2}V*F}{R*T}\right)}$ and *A* from the four closed states (*C_i_*, *i* = 0, …, 4) to the open (O) state and the backward rates $\beta \;=\beta_{1}\;\cdot \;{\text{exp}}^{-\left(\frac{\beta_{2}V*F}{R*T}\right)}$ and *B* from the open (O) to the closed states *C_i_* (Figure 2B). The parameters were derived from experiments performed in TagRFP‐K_v_1.3 transfected HEK293 cells to model the activation and deactivation, respectively (Figure S2A–C, Supporting Information) using hybrid particle swarm optimization model bounds based on the description of K_v_1.3 in ref. [26] for the set of parameters [α_1_,β_1_,*m*, *n*, *A*, *B*, *N*].The transition rates were finally set to

(7) $$\begin{array}{c} \begin{array}{l} \alpha_{1}=\;281.4283\;s^{-1},m\;=\frac{R\;\cdot \;T}{\alpha_{2}\;\cdot \;F}\;=\;0.0255\;V,\;\beta_{1}=\;120\;s^{-1},\\ n\;=\frac{R\;\cdot \;T}{\beta_{2}\;\cdot \;F}\;=\;0.5623\;V,\;A\;=\;4.4432e4\;s^{-1},B\;=9.6566e3\;s^{-1} \end{array} \end{array}$$

The number *N* of channels and thus the overall conductivity *G*_M_ (*V*,*t*) = *G*_*Kv*1.3_(*V*,*t*) is used to adjust the number of active ion channels in each individual cell.^[^ ^30^ ^]^ The simulated K_v_1.3 channel dynamics correlated well to functional data for the activation, short activation, and step‐wise activation protocols (Figure S2A–C, Supporting Information).

### 3.3 Equivalent Clamp Voltage

We first compared the effect of OEPC stimulation to the stimulus *V*_C_′ provided by the patch‐clamp amplifier in voltage‐clamp mode. To obtain an idea of the currents generated by the OEPC device, we computed the equivalent clamp voltage *V*_C_′ that would be necessary to measure the same current *I*_out_ at the patch‐clamp output in voltage‐clamp mode without adding a stimulus inside the cleft ( *V*_in_ = 0), by feeding the measured current into the model and computing $V_{C}^{\prime }$ as output. OEPC stimulation in voltage‐clamp mode using the maximum light intensity of 13 mW mm^–2^ corresponds to a biphasic clamp voltage stimulus with an average maximum voltage shift of about +51.2 mV (±0.3 mV), a stable plateau about 11.9 mV (±0.8 mV), and a minimum peak of about ‐38 mV (±0.01 mV) (Figure 2D). The membrane voltages needed to provoke these currents are provided in Figure S2E (Supporting Information) yielding a similar voltage profile as $V_{C}^{\prime }$ (Figure 2D). We could further determine the total conductance of K_v_1.3 channels in this cell to be *G*_M_ = *G*_Kv1.3,TagRFP_ = 1.44 × 10^−7^S at a clamped voltage of *V*_C_ = 40 mV. At a clamped voltage of *V*_C_ = − 50 mV, the model shows for example a maximum increase in K_v_1.3 conductance ( *G*_M_ = *G*_Kv1.3,TagRFP_) of the attached membrane of about 46 nS. The channels would not conduct without light mediated OEPC stimulation at this level. Figure 2E shows the increase in K_v_1.3 conductivity of the attached membrane. These simulations correspond well to our experimental finding (Figure 1G), where we could also observe a shift of the conductivity over the whole cell during illumination.

### 3.4 OEPC Stimulus in Voltage‐Clamp Mode

In the next step, we compared the simulated output currents *I*_out_ of the two domain model with the clamp voltage *V*_C_′ and the OEPC stimulus *V_J_* in the cleft as inputs to the measured currents adjusting the conductivity *G*_M_ = *N* · *g*_Kv1.3,TagRFP_ · *P*_o_(*t*) for each specific cell using the parameter *N* for the number of channels (*P*_o_ describes the fraction of channels in the open state, *N* is used to adjust the model to the measured current for each cell individually). We could show that the light‐induced currents of ion channel activation in a single cell (Figure 2G) correspond well to the modeled ones (Figure 2F,H). The simulated ion channel current in combination with the OEPC stimulation model and the cell geometry, allowed to determine channel opening in the attached and free membrane. It is of note that the ion channels in the attached membrane predominantly contributed to light induced K_v_1.3 channel conductivity. The resulting membrane voltages of the attached and free membrane are depicted in Figure 2I.

The simulated attached membrane voltage (orange trace) determines a first depolarized peaking and, as soon as the light is switched off, this part of the membrane is hyperpolarized and turns back to the clamped voltage (Figure 2I). The free membrane (blue trace), on the other hand, is only slightly affected by the illumination. It is of note that the rather high membrane perturbation of the attached membrane is in part due to the amplifier which is trying to keep *V*_C_′ at the same level as the command voltage *V*_C_.

### 3.5 OEPC Stimulus in Current Clamp Mode (*I* **=** 0)

Clamping the pipette to a specific voltage is necessary to determine the kinetics of voltage‐gated ion channels and to show the early channel opening in response to OEPC stimulation. However, the more natural behavior of the cell can be observed in current‐clamp mode, where the voltage from the inside of the cell relative to the bath electrode is measured (*V*_I_). To model this situation, the amplifier in Figure 2A can be replaced by a voltage follower, which results in very low currents flowing to the patch system. Figure S2F (Supporting Information) shows the voltage *V*_I_ measured by the patch system relative to the bath electrode and the simulation result. Figure S2G (Supporting Information) shows the respective voltages across the attached and the free membrane. It can be observed that also in this setup, the attached membrane first depolarizes and hyperpolarizes as soon as the light is switched off.

### 3.6 Neuronal Cell Stimulation

We evaluated next, whether an OEPC‐induced membrane depolarization exceeds the threshold to induce AP firing. We used similar OEPC devices as for the light‐dependent K_v_1.3 channel activation, but with PEDOT:PSS covering the whole surface. Recent experiments have clearly demonstrated that PEDOT:PSS modification of the whole device lowers interfacial impedance and also allows the maximum membrane polarization to be sustained over the whole illumination time.^[^ ^16^ ^]^ We investigated optoelectronically controlled neuronal signaling in a primary culture of neuronal‐glial mixture extracted from hippocampi of postnatal rats (P0–P1). This in vitro model system aims to mimic the trophic support provided by glial cells to obtain a neuronal population as viable as possible. Cells were cultured for two to three weeks on OEPC devices spin‐coated with a thin film of PEDOT:PSS (Figure S3A, Supporting Information) prior to recordings to allow for a mature in vitro network. The neurons grew well on the PEDOT:PSS layer coated OEPC device (Figure S3B, Supporting Information) with typical interconnections and neural clustering similar to in vitro cultures on poly‐Lys coated glass coverslips. The neuronal‐glial culture showed no morphological abnormalities, and the cell viability was not significantly varied compared to the control groups cultivated on plain glass coverslips (Figure S3C, Supporting Information). These results in primary cells, together with the findings on cell line, clearly show that the herein described OEPC devices were stable and biocompatible for long‐term in vitro applications.

**Figure 3:**
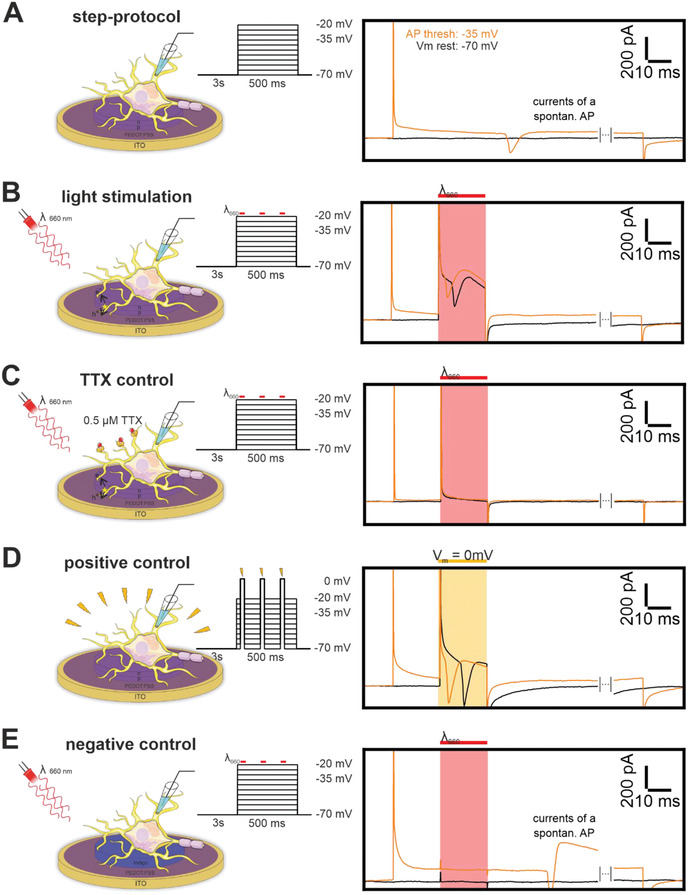
Excitatory currents of hippocampal primary neurons on PEDOT:PSS modified OEPCs measured with the perforated patch‐clamp technique in voltage‐clamp mode. A) Schematic set‐up (not to scale) and traces of the step protocol at *V*_c_ ‐70 and ‐35 mV showing excitatory currents for a spontaneous AP at threshold potentials; representative cellular current profile (*n* = 17). B) Same step protocol like in A with a 20 ms laser light pulse (highlighted in red); traces from ‐70 mV resting membrane and ‐35 mV threshold potentials show a representative excitatory current profile (*n* = 12) with a light‐induced positive capacitive peak, followed by the characteristic excitatory current profile, and a negative capacitive transient. C) Same protocol like in B with neurons perfused in an extracellular solution containing 0.5 × 10^–6^ m tetrodotoxin to prevent AP firing by blocking Na^+^ channels; traces from ‐70 mV resting membrane and ‐35 mV threshold potential show light induced capacitive transients but no excitatory current profile (*n* = 8). D) Step protocol for a representative cellular current profile (*n* = 15) with an additional depolarization step to 0 mV for 20 ms without a laser light pulse; traces from ‐70 mV resting membrane and ‐35 mV threshold potential show a positive capacitive transient from clamping the membrane potential to 0 mV, followed by the characteristic excitatory current peak, and a negative capacitive transient from clamping the membrane potential to the command voltage. E) Schematic set‐up of a representative OEPC heat control (Indigo, *n* = 6) and traces of the step protocol at *V*_c_ ‐70 and ‐35 mV showing little capacitive transients and no light induced but spontaneous excitatory currents.

In patch‐clamp experiments, we initially assessed the resting membrane potentials of individual cells at *I* = 0 pA: ‐64 ± 2.8 mV and the threshold potential for spontaneous APs: ‐34.4 ± 3.5 mV by current injection in current‐clamp configuration. Neurons that responded with AP firing were voltage clamped at a typical resting membrane potential of ‐70 mV for 3 s to avoid inactivation of Na^+^ channels. Membrane depolarization was then induced by a stepwise increase of the holding potential by +Δ10 mV for 500 ms. No AP currents were recorded between resting membrane potential from ‐70 to ‐40 mV (**Figure** **3**A). At the AP threshold potential, fast spontaneous excitatory inward currents started to appear, followed by a longer‐lasting outward current (Figure S4A, Supporting Information, orange trace).

Next, we voltage‐clamped neurons at ‐70 mV and illuminated the OEPC device for 20 ms using a red diode laser with an intensity of 26 mW mm^–2^. In addition to the capacitive current peaks typical for switching the light on and off, we observed the characteristic excitatory currents of an AP. Importantly, we now observe excitatory currents evoked by illumination even at resting membrane potentials of ‐70 mV (Figure 3B). When clamping the membrane to a more positive potential (e.g., ‐60 mV or ‐50 mV) the excitatory inward current shifted closer to the positive capacitive transient, suggesting that the threshold for AP firing was surpassed slightly earlier (Figure S4B, Supporting Information). Certain neurons generated multiple excitatory currents during one illumination pulse (Figure S3D, Supporting Information). To specifically interfere in membrane depolarization of voltage‐gated Na^+^ channels, we subsequently perfused the neuronal cultures on functional OEPC devices with an extracellular solution containing 0.5 × 10^–6^ m Tetrodotoxin (TTX) subsequent to firing APs from voltage‐clamp protocol as in Figure 3B. With TTX incubation, we observed the same capacitive peaks induced by light but no subsequent excitatory currents (Figure 3C and Figure S4C, Supporting Information). Therefore, we show that the Na^+^ channel inhibitor TTX blocks efficiently light‐induced AP firing. We then subtracted the current traces of the same cells before (Figure S5A, Supporting Information) and after TTX perfusion (Figure S5B, Supporting Information) to isolate the excitatory AP currents. In addition, to small light artifacts, we were now able to visualize the characteristic voltage‐gated Na^+^ inward current followed by the outward K^+^ current during AP firing (Figure S5C, Supporting Information) directly. In further control experiments, we mimicked our light protocol with an additional voltage depolarization step to 0 mV with the same length as the 20 ms light pulse (Figure 3D and Figure S4D, Supporting Information). This additional membrane depolarization mimicked excitatory currents similar to our previous millisecond light provoked currents (Figure 3B,D and Figure S4B,D, Supporting Information). To rule out photothermal activation and light artifacts caused by the illumination setup, we repeated previous experiments with a device having an indigo layer in place of the PN photopixel. The indigo variant has a similar absorption spectrum but cannot undergo charge separation during the light pulses as it efficiently converts the absorbed light into heat^[^ ^31^ ^]^ and hence does not generate photocapacitive charging (Figure 3E and Figure S4E, Supporting Information). Only small transients were induced by switching the light on or off without stimulating any excitatory currents at the resting membrane potentials (‐70 mV). Neuronal firing on the indigo layer appears just at the threshold potential (‐35 mV) and resembles the firing on plain glass which was also independent of the illumination protocol.

**Figure 4:**
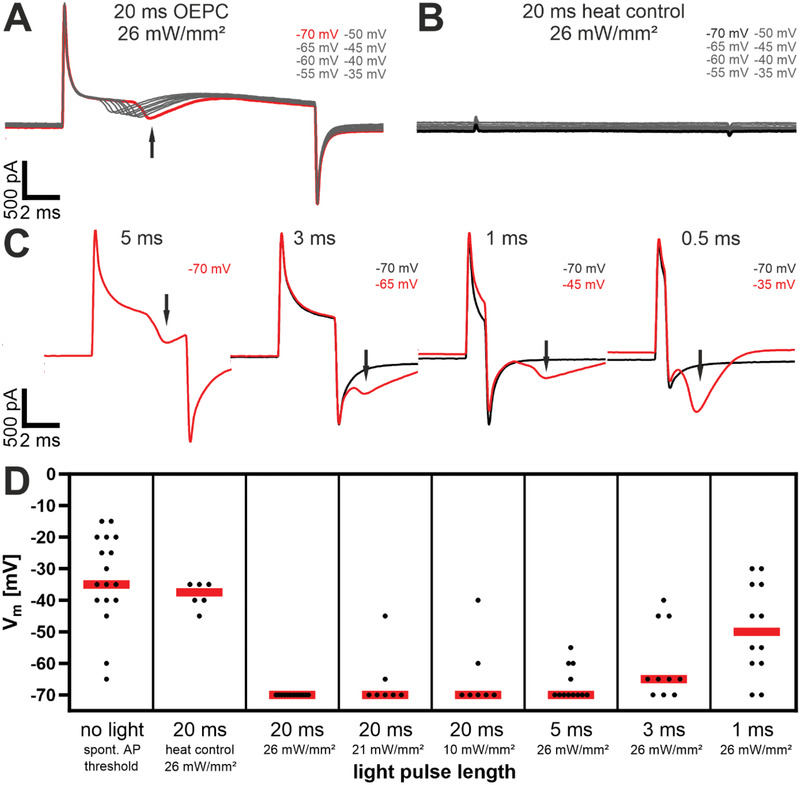
Comparison of different laser pulse lengths and intensities with the corresponding holding command voltage for light stimulated excitatory neuronal currents; measured with the perforated patch‐clamp technique in voltage‐clamp mode. A) Traces of a step protocol from a single representative neuron during a 20 ms light pulse; the red trace shows the most negative holding potential that is necessary for inducing ion channel gating upon light stimulation; the arrow shows the characteristic excitatory currents of an AP (*n* = 12). B) Traces of a step protocol from a single representative neuron (*n* = 6) on a heat control device during a 20 ms light pulse. C) Traces of a step protocol from a single neuron during different light pulse lengths; the black traces show a photocurrent without voltage‐gated channel activation at holding potentials of ‐70 mV (*n* = 12). D) Overview of multiple experiments with each dot representing a neuron (*n* ≥ 6 per group) and the median values (red line) of the clamped membrane holding potential necessary for light‐induced AP activation; “no light”‐column on the left shows varying threshold potentials for every single neuron.

In the next step, we evaluated the efficiency of AP firing depending on the light pulse length and intensity as well as on the holding membrane potential. With our previous settings, the maximum light intensity of 26 mW mm^–2^ and a length of 20 ms yielded 100% efficacy in AP stimulation at ‐70 mV. This protocol evoked firing at the resting membrane potential as well as on more positive holding potentials (‐65–‐20 mV) in all 12 neurons (**Figure** **4**A,D). Also, at lower light intensities (21 and 10 mW mm^–2^), the overall majority (22 out of 26 neurons) activate excitatory currents at all clamped membrane potentials during a 20 ms light pulse. Only two neurons in each condition failed at resting membrane potential (‐70 mV) to fire APs. They show their first excitatory currents rising at slightly more depolarized membrane potential (Figure 4D). Next, we investigated the pulse length at the maximal light intensity that is required for neuronal firing. Within a 5 ms light pulse, 8 out of 12 neurons activated at ‐70 mV (black arrow, Figure 4C) whereas, for 4 neurons, excitatory currents arose at more depolarized membrane potentials (‐65 mV to ‐55 mV, Figure 4D). At shorter pulses, the median for single neuron AP activation was induced by thresholds that were significantly lower than those of spontaneous APs (Figure 4D). We observed for 20 and 5 ms light pulses that the ion channel activation (black arrows Figure 4A,C) is within the photogenerated currents while for shorter illumination periods (3, 1, and 0.5 ms) the excitatory currents developed after switching the light off (Figure 4C). These characteristics are also present in current‐clamp measurements in the next paragraph. Neurons attached to heat control devices showed no AP activation during the light pulse (Figure 4B) and a similar onset of spontaneous APs like the nonilluminated neurons (Figure 4D). In summary, all investigated illumination protocols showed a significant generation of AP firing at or close to the resting membrane potential which is important for a physiological application in vivo.

**Figure 5:**
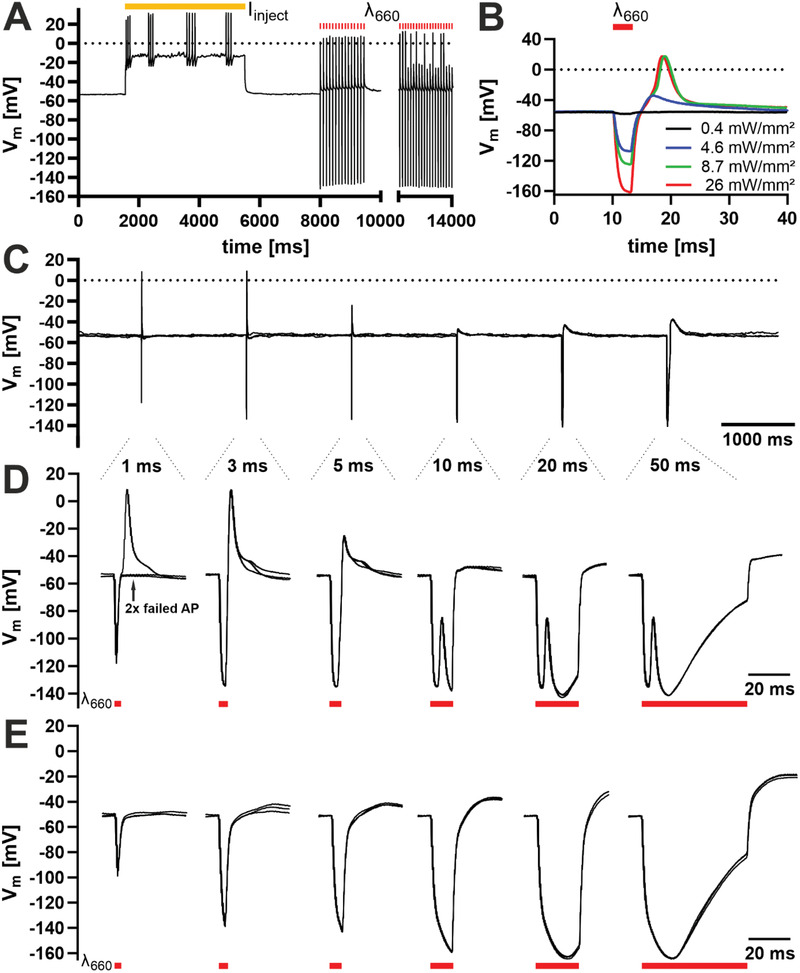
Photocapacitive generated action potentials of neurons DIV14‐21 on PEDOT:PSS spin‐coated OEPCs measured with the perforated patch‐clamp technique in current‐clamp mode. A) Excitability test of a single, representative neuron (*n* = 3) through current injection (yellow bar) between *t* = 2–6 s reaching the threshold potential for spontaneous trains of APs; *t* = 8–10 s and *t* = 12–14 s show the membrane potential during a train of 3 ms laser light pulses and evoked or failed APs. B) A representative neuron (*n* = 3) with the membrane potential during a single 3 ms light pulse *t* = 10–13 ms (photogenerated cathodic stimulation artifact peaking at ‐160 mV) followed by a failed AP at 0.4 (black) and 4.6 mW mm^–2^ light intensity (blue) not reaching the threshold potential; *t* = 13–20 ms shows triggered AP at light intensities of 8.7 mW mm^–2^ (green) or higher. C,D) The membrane potential and light‐induced APs of a single representative neuron (*n* = 5) during different light pulses varying in length from 1 to 50 ms; overlay of three traces. E) Membrane potential of another neuron not responding to the light pulses; overlay of three traces.

Finally, we recorded light‐mediated neuronal responses to OEPCs directly in current‐clamp measurements. As initial control, we used current injections and clamped the membrane to the neurons’ threshold potentials which induces trains of spontaneous APs with peak voltages of around +20 mV (**Figure** **5**A, 2–6 s). To investigate AP firing at resting membrane potentials, we set *I* = 0 pA and applied repetitive 3 ms light pulses. This light pulse series is directly visible by short photogenerated negative voltage peaks. As OEPC generates a cathodic stimulation, the generated light‐induced electric potential is visible as a negative voltage peak between the patch pipette and the bath reference electrode. We refer to these negative voltage peaks here as cathodic stimulation artifacts, as they do not record voltage time‐courses generated by the cell membrane. Hence, these peaks are similarly observed in the electrolytic bath when no cell is attached to the patch pipette. After the cathodic stimulation artifact an AP mediated depolarization of the neurons was detected. While the first train of 3 ms light pulses had an efficacy of 100 % to induce APs on single pulses (Figure 5A, 8–10 s), a second train showed partial failures in AP generation due to channel inactivation (Figure 5A, 12–14 s). Next, we tested different light intensities for 3 ms pulses (Figure 5B). During OEPC illumination we recorded again cathodic stimulation artifact peaks of up to ‐160 mV with maximum light intensity (red trace 26 mW mm^–2^) followed by a time‐correlated and fast upstroke from ‐60 to +20 mV after the light pulse (Figure 5B). Decreasing the light intensity correlated with a smaller cathodic stimulation artifact peak, while the AP‐mediated depolarization peak remained constant between 26 and 8.7 mW mm^–2^ light intensity. Only at low intensities (Figure 5B, 4.6 mW mm^–2^ blue curve), the initiation of an AP failed. We compared illumination‐generated AP firing on OEPCs with the spontaneous APs triggered by current injection (Figure S6A, Supporting Information) and determined similar slopes for both AP time‐courses during the depolarization and the hyperpolarization phase (Figure S6B, Supporting Information, green).

In the last experiment, we investigated the time‐course of light‐triggered AP firing of neurons and the intertwined cathodic stimulation artifact peak that is additionally generated by the OEPC. For very short light pulses, the AP generation followed directly after the cathodic stimulation artifact peak (Figure 5C). Failed AP time‐courses of the same neuron clearly showed only the fingerprint of OEPC‐mediated cathodic stimulation artifact (Figure 1, 5, 1 ms). Longer illumination protocols (10–50 ms) extended the cathodic stimulation artifact peak. Here, the AP‐mediated depolarization is visible as a short depolarizing peak within the cathodic stimulation artifact in the time course (Figure 5D). We observed AP firing caused by the light pulse in all active neurons. To illustrate only the cathodic stimulation artifact peak, we plotted the time course of a few neurons that were generally not active. In these cells, the time course of the cathodic stimulation artifact peak was similar as in Figure 5D, yet without the additional AP mediated depolarization (Figure 5E). Specifically, for 5 ms pulses the intertwined voltage fingerprints of cathodic stimulation artifact peak and AP generation partially cancel each other, hence only a small depolarizing AP peak was observed (Figure 5D). These findings are consistent with our voltage‐clamp experiments and show that light pulses with different illumination times efficiently generate APs.

## 4 Discussion

The present study demonstrates that OEPC devices induce fast membrane depolarizations sufficient to activate voltage‐gated ion channels in mammalian cells and consequently trigger APs in cultured primary neurons upon millisecond light pulses. OEPCs are relatively new thin‐film devices that can act as light pulse driven extracellular stimulation electrodes.^[^ ^15^ ^]^ They mimic traditional biphasic cathodic leading charge‐balanced stimulations,^[^ ^32^ ^]^ and have been successfully validated for the stimulation of retinal tissues as well as peripheral nerves.^[^ ^14^, ^18^ ^]^ Understanding their action at the level of small, single cells, has been very limited to date. For this reason, we have studied small, nonexcitable cells, as well as cultured neurons.

As a model for voltage‐gated ion channel activation by OEPC stimulation, we used K_V_1.3 channels expressed in HEK cells. K_v_ channels offer the advantage of slower gating kinetics compared to sodium channels and a reduced inactivation rate. Their kinetics is slow enough to enable recording of channel activation overlaid together with the stimulation artifact originating from the OEPC. Hence, patch‐clamp recordings directly visualized photoinduced channel stimulation. Upon light stimulation of transfected HEK cells on the OEPC surface, measured K_v_1.3 conductance‐voltage curves shifted by ≈27 mV. This is clear evidence of net cell membrane depolarization driven by the light pulse. The underlying time‐ and position‐dependent membrane potential polarization by OEPCs is, however more complex. To understand the interplay between different parts of the cell membrane, OEPC extracellular stimulation, and the patch‐clamp amplifier, we defined an OEPC stimulation model including a detailed consideration of the K_v_1.3 channel kinetics. We base this on the two‐domain model for single cell extracellular electrode stimulation proposed by Fromherz,^[^ ^21^ ^]^ which separates the cell membrane into one part attached to the electrode and another part, which is considered to be free in the medium. The reduction to this model provides valuable information about ion channel activation caused by external stimulation methods.

To obtain comparable results to the experiments and easier interpretation, we first estimated the voltage clamp stimulation by the patch‐clamp amplifier that would lead to the same current as induced experimentally by OEPC stimulation. These simulations show that the conductivity of K_v_1.3 increases to approximately the same level as with a 20 mV higher current stimulation pulse, which supports our experimental findings. In the next step, we determined the membrane voltages of the free and the attached membrane that would be necessary to produce the measured current upon light stimulation. In accordance with previous research, we could show that the attached membrane is first depolarized upon illumination and hyperpolarized as soon as the light is switched off. The free membrane is mainly controlled by the patch‐clamp amplifier and thus, only slightly affected and polarizes in the opposite direction. Our simulations determined that the OEPC stimulation mainly affected the attached membrane within the first milliseconds of a light pulse. The large depolarization of the attached membrane is partially caused by the complex interplay of the patch‐clamp amplifier in voltage‐clamp mode which is trying to keep the inside of the cell at the predefined value, the external stimulation through OEPC activation and a relatively small area of the cell being in closer contact to the device surface. The patch‐clamp measurement method, however, is limited to resolve this difference in space since one can only determine the sum of currents over the entire cell membrane or voltages over the access resistance and the entire membrane related to the bath electrode.

The voltage‐clamp mode is a valuable tool to study the kinetics of voltage‐dependent ion channels, however, measuring in current clamp (*I* = 0) mode is a more natural situation for the cell. Thus, we adapted the OEPC stimulation model to current‐clamp mode and could show that our modeling results fit the measurements well. In current‐clamp simulations, we could observe a lower membrane voltage perturbation of the attached membrane, which is due to the omission of the amplifier's feedback loop. Consequently, the free membrane is more hyperpolarized in the beginning and depolarizes as soon as the light is switched off.

The time courses of membrane polarization of the free and attached membrane were obtained in a nonexcitable cell line, yet presented a strong mechanistic basis to investigate OEPC mediated membrane polarization for AP firing. The fast light‐dependent membrane depolarization of the attached membrane was subsequently investigated in hippocampal neurons. Here the OEPC could clearly evoke AP firing. Both voltage clamp and current clamp revealed that light pulses of 3 ms or longer induced highly efficient and repetitive AP firing. In voltage‐clamp experiments, APs were generated from the resting potential of ‐70 mV. When neurons were clamped to less negative potential, we observed that light pulse generated APs appear slightly earlier due to a lower voltage change needed to surpass the threshold.

The behaviors of the voltage‐gated channels in HEK cell, and the stimulation of APs in neurons, conform to the conclusion that OEPCs act as a photovoltaic‐driven extracellular stimulation electrode. OEPCs produce a capacitive charging current of cathodic polarity when the light pulse begins, resulting in a discharging current of opposite polarity when the light pulse ends. In this way, an OEPC transduces an optical on/off square pulse into a biphasic capacitive cathodic‐leading pulse.

In contrast to our OEPCs that reach AP thresholds with single light pulses, most external stimulations rely on multipulse protocols. Pioneer work in capacitive stimulation experiments featured wired capacitors near the cells’ surface. The authors used a train of voltage pulses stimulating the opening of Na^+^ channels and therefore a gradual membrane depolarization with every step that sums up until the threshold potential for AP firing was reached.^[^ ^33^ ^]^ The actual number of pulses necessary for activating a neuron is also dependent on the resting membrane potential of the respective cell. Organic bulk‐heterojunction photocapacitors, based on donor copolymer PTB7‐Th and an acceptor small molecule PC71BM or similar compounds achieved only single APs upon continuous trains of repetitive 1 ms light pulses. However, these stimulation protocols required up to 100 ms to generate a single AP.^[^ ^34^, ^35^ ^]^ Other reports established photostimulation based on (poly(3‐hexylthiophene‐2,5‐diyl)) P3HT surfaces that generated AP firing and even restored light sensitivity in a blind rat retina.^[^ ^36^, ^37^, ^38^ ^]^ The mechanism of stimulation in these types of systems remains unclear and a subject of debate.^[^ ^39^ ^]^ Other materials based on silicon^[^ ^40^ ^]^ or gold nanoparticles^[^ ^41^ ^]^ use photothermal heating effects for neuronal excitation. Especially the thermocapacitive stimulation is a well‐described system^[^ ^42^ ^]^ and can achieve localized^[^ ^41^ ^]^ and less invasive^[^ ^43^ ^]^ cell stimulation.

## 5 Conclusion

Our work validates the efficacy of OEPCs for stimulation of single cells, and lays a firm mechanistic understanding of how this form of extracellular stimulation operates. Based on our findings, OEPCs enable novel optically driven in vitro experimental protocols, to study samples in a wireless manner by inducing action potentials at the single‐cell scale. While clearly a useful experimental tool for in vitro biophysics, we believe that our results also inform the use of these devices for in vivo contexts.

## Conflict of Interest

The authors declare no conflict of interest.

## Author Contributions

R.S., T.S., T.R., and E.D.G. conceived of the ideas, supervised the project, interpreted the data, and wrote the manuscript. T.S. prepared the original draft of the paper and performed patch clamp experiments and analyzed the data. T.S. and L.W. performed cell viability. O.T., S.P., T.S., H.B., and S.B. performed cell preparation, cultivation, cloning, and biochemical work. M.J. produced and evaluated the OEPC devices. K.K. and T.S. performed SEM experiments. T.S. and M.N. performed fluorescence experiments and 3D deconvolution. T.R. and V.Đ. conducted mathematical modeling and simulation. M.U., R.M., S.S., C.B., and G.L. contributed to the study design, revised the manuscript, and supported data analysis together with all other authors.

## Supporting information

Supporting Information

## Data Availability

The data that support the findings of this study are available from the corresponding author upon reasonable request.
