# Supplementary material for: Light Stimulation of Neurons on Organic Photocapacitors Induces Action Potentials with Millisecond Precision
Source: Adv Mater Technol. 2022 Mar 18;7(9):2101159. doi: 10.1002/admt.202101159 (PMC10097427; doi:10.1002/admt.202101159)
Supplement: Supplementary file 1 — Supporting Information [file ADMT-7-2101159-s001.pdf]

ADVANCED MATERIALS TECHNOLOGIES

Supporting Information

for *Adv. Mater. Technol.,* DOI: 10.1002/admt.202101159

Light Stimulation of Neurons on Organic Photocapacitors Induces Action Potentials with Millisecond Precision

Tony Schmidt, Marie Jakešová, Vedran Đerek, Karin Kornmueller, Oleksandra Tiapko, Helmut Bischof, Sandra Burgstaller, Linda Waldherr, Marta Nowakowska, Christian Baumgartner, Muammer Üçal, Gerd Leitinger, Susanne Scheruebel, Silke Patz, Roland Malli, Eric Daniel Głowacki, Theresa Rienmüller,* and Rainer Schindl**

Supporting Information

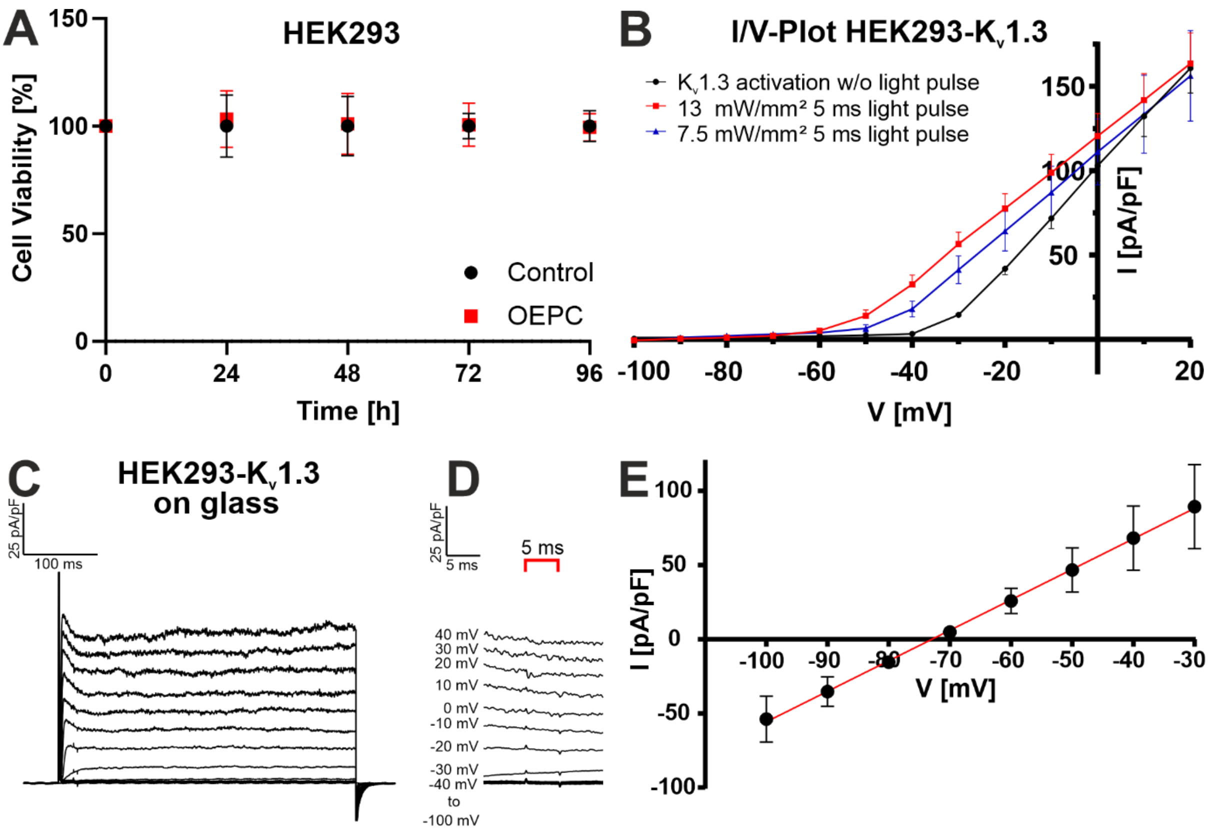

Fig. S1 **Electrophysiology of K_v1.3 channels heterologously expressed in HEK cells on OEPCs.** (A) Cell viability of HEK293 cells (+SD) on OEPCs (red) compared to the respective control group seeded on poly-D-lysine coated glass coverslips (black) with n = 6. (B) Current/voltage-plot of whole-cell voltage clamped HEK293-K_v1.3 cells on OEPCs at different light intensities 13 mW/mm² (n=17), 7.5 mW/mm² (n=10) and no light stimulation (n=17). (C) Whole-cell voltage clamped HEK293 cells on glass (D) with zoom into the 5 ms LED pulse of 13 mW/mm² at 660 nm. (E) Measured reversal potential of the transfected K_v1.3 ion channel (n=7).

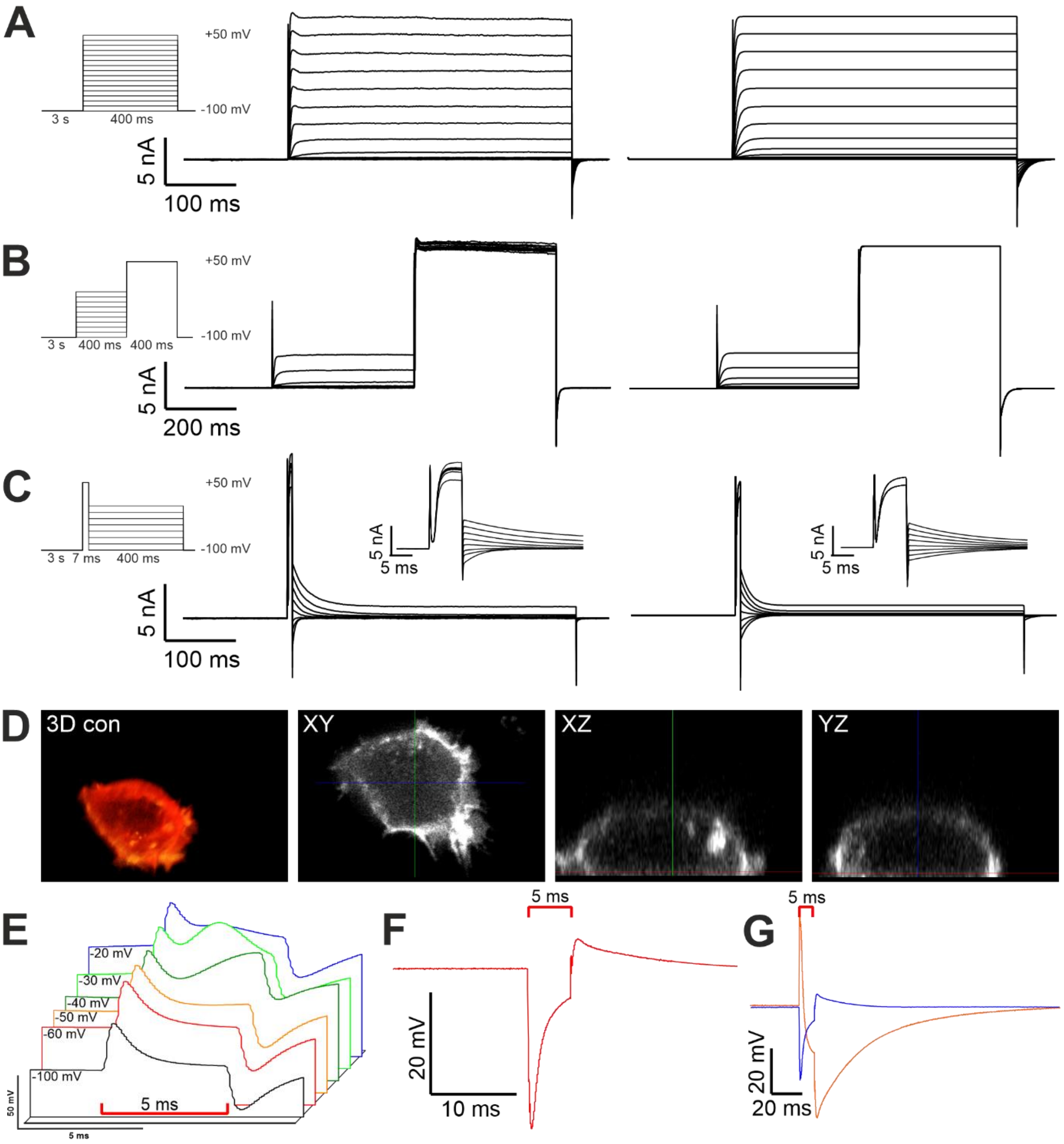

Fig. S2 **K_v1.3 currents in voltage-clamp recordings in comparison to simulated current time-courses.** (A) Patch clamp voltage step protocols (left panel) and recorded (middle panel) as well as simulated current time-courses (right panel) to obtain ion channel kinetics including an activation protocol, (B) stepwise activation and (C) short activation and deactivation (each n = 23) with zoom into traces as inset. In this example the cell parameters were found to be $R_M = 325.67\ \mathrm{M\Omega}, C_M = 30\ \mathrm{pF}, R_S = 2\ \mathrm{M\Omega}, R_{\mathrm{seal}} = 1.6957\ \mathrm{G\Omega}, R_J = 10\ \mathrm{M\Omega}$, $R_E = 7.69\ \Omega$ [12], $C_J = 1\ \mathrm{mF}$ [15]. The conductivity was set to $G_M = N \cdot g \cdot P_O = 2.3166\mathrm{e} - 07 \cdot P_O$ (D) 3D convolution of an example HEK293 cell attached 3 h after seeding. (E) Simulated equivalent membrane voltage of the entire cell. (F) Modeled ion channel voltage time-course based on patch clamp experiments in current-

clamp recordings. (G) Simulated voltage time-courses across the attached (orange) and free (blue) membrane in simulated current-clamp mode.

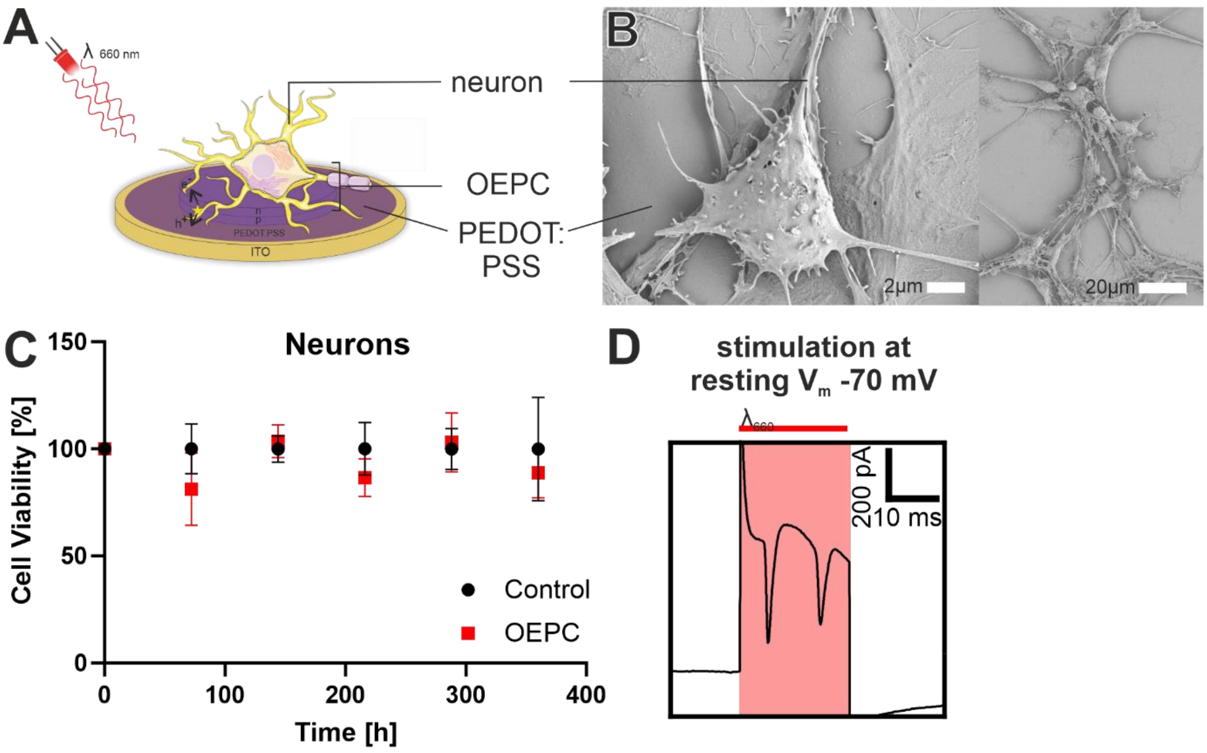

Fig. S3 Cell viability of neurons on OEPCs. (A) Schematic representation and (B) SEM imaging of interconnected neurons DIV14-21 with glia on OEPCs spin-coated with PEDOT:PSS. (C) Cell viability of neurons (+SD) on OEPCs (red) compared to the respective control group seeded on poly-D-lysine coated glass coverslips (black) with n = 10. (D) Ionic currents of two APs in hippocampal neurons at DIV14-21 on PEDOT:PSS spin-coated OEPCs measured with the perforated patch clamp technique in voltage clamp mode. Step protocol as in Figure 3B clamped to V_c -70 mV and a laser light pulse highlighted in red.

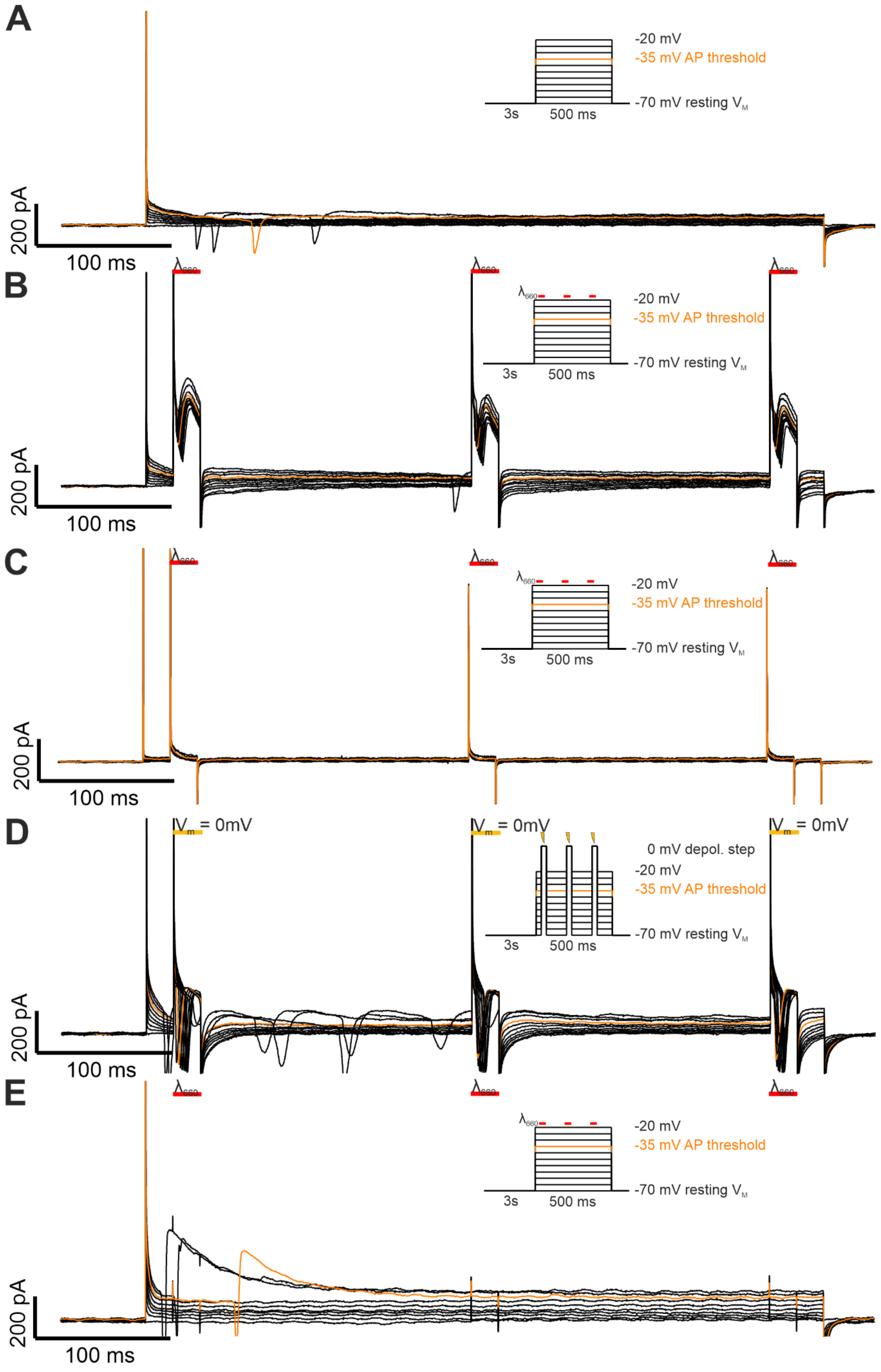
A
-20 mV
-35 mV AP threshold
-70 mV resting V_M
3s
500 ms
200 pA
100 ms
B
λ_{660}
-20 mV
-35 mV AP threshold
-70 mV resting V_M
3s
500 ms
200 pA
100 ms
C
λ_{660}
-20 mV
-35 mV AP threshold
-70 mV resting V_M
3s
500 ms
200 pA
100 ms
D
V_m = 0mV
0 mV depol. step
-20 mV
-35 mV AP threshold
-70 mV resting V_M
3s
500 ms
200 pA
100 ms
E
λ_{660}
-20 mV
-35 mV AP threshold
-70 mV resting V_M
3s
500 ms
200 pA
100 ms

Fig. S4 **Ionic currents of neurons DIV14-21 on PEDOT:PSS spin-coated OEPCs measured with the perforated patch clamp technique in voltage clamp mode.** (A) Step protocol with a membrane holding potential between -70 mV and -20 mV in Δ5 mV steps; traces show the excitatory currents for spontaneous APs above the threshold of -35 mV for this neuron. (B) Same step protocol like in A with three 20 ms laser light pulses at the beginning, in the middle and at the end of the depolarization step; at -70 mV resting membrane potential and all subsequent command voltages, traces show a light-induced change with a positive capacitive peak, followed by the characteristic current profile evoked by an AP and a negative capacitive transient. (C) Same step protocol as in B with neurons perfused in an extracellular solution containing 0.5 µM tetrodotoxin. Traces show light induced capacitive transients but no excitatory current profile. (D) Step protocol between -70 mV and -20 mV in Δ5 mV steps with a threshold reaching depolarization step to 0 mV for 20 ms instead of a laser light pulse; traces show a positive capacitive transient from clamping the membrane potential to 0 mV, followed by the characteristic current profile evoked by an AP and a negative capacitive transient from clamping the membrane potential to the command voltage. (E) Same step protocol as in B with an OEPC heat control (Indigo) showing little capacitive transients and no light induced but spontaneous excitatory currents.

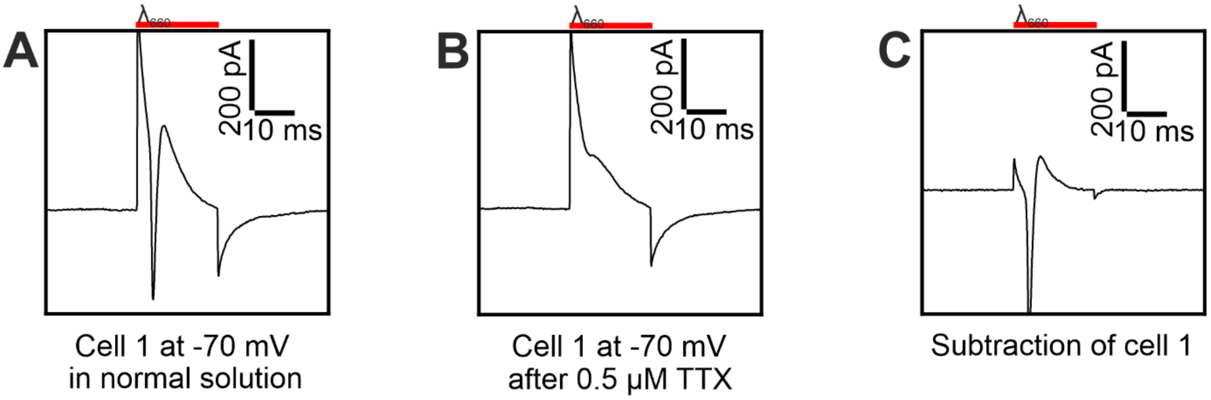

Fig. S5 **Ionic currents of neurons DIV14-21 on PEDOT:PSS spin-coated OEPCs measured with the perforated patch clamp technique in voltage clamp mode.** (A) Step protocol as in S4B at the command voltage of -70 mV during a 20 ms light pulse; the trace shows the excitatory current of an AP between the capacitive transients. (B) The same cell perfused with an extracellular solution containing 0.5 µM tetrodotoxin; the trace shows similar light induced transients without the excitatory current due to the inhibition of Na^+ channels necessary for AP firing. (C) Difference between traces from A and B showing the excitatory current profile of an AP together with smaller light dependent artefacts when the light is turned on and off.

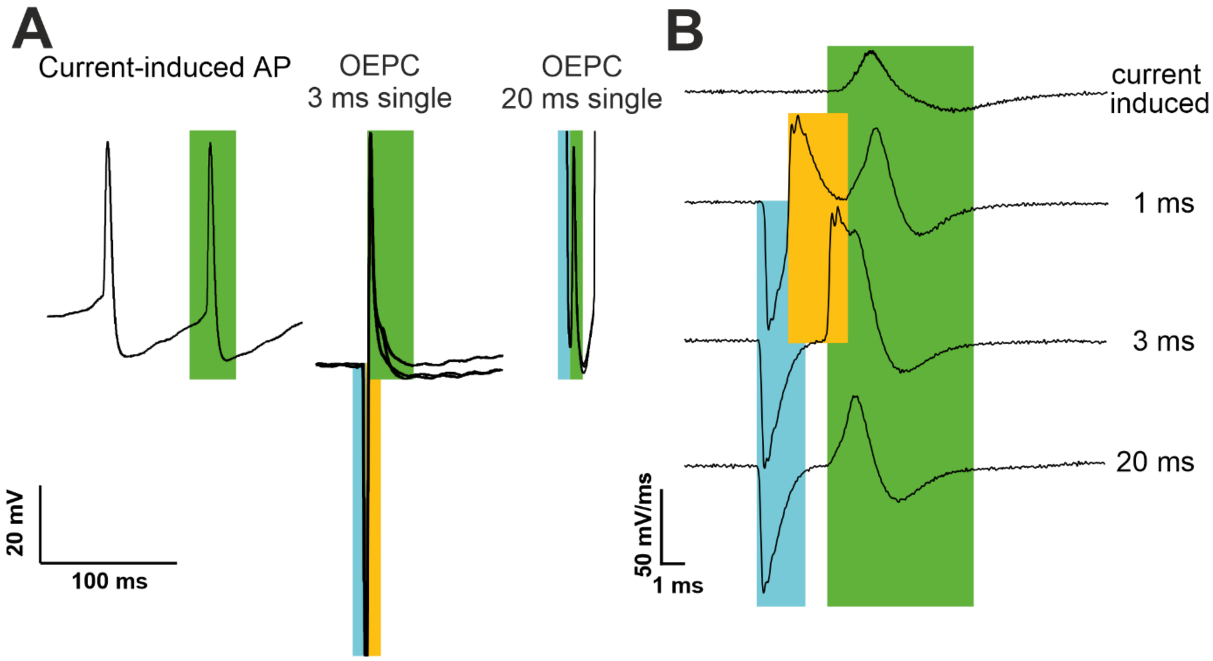

Fig. S6 **Membrane potential of neurons DIV14-21 on PEDOT:PSS spin-coated OEPCs measured with the perforated patch clamp technique in current-clamp mode.** (A) Traces of current injected APs, and light induced APs of 3 ms or 20 ms at I = 0 pA. (B) dV/dt vs t plot and the slope of the rising and falling time-courses during AP firing and light stimulation; blue negative slope and yellow positive slope of the photogenerated cathodic stimulation artefact, green slope of the falling and rising times of the AP.

Model equations

The HMM for the TagRFP-K$_v$1.3 channel can be described by the following equation

$$\frac{\mathrm{d}\mathbf{P}(t)}{dt} = \begin{bmatrix} -4\alpha & \beta & 0 & 0 & 0 & 0 \\ 4\alpha & -3\alpha-\beta & 2\beta & 0 & 0 & 0 \\ 0 & 3\alpha & -2\alpha-2\beta & 3\beta & 0 & 0 \\ 0 & 0 & 2\alpha & -\alpha-3\beta & 4\beta & 0 \\ 0 & 0 & 0 & \alpha & -4\beta-A & B \\ 0 & 0 & 0 & 0 & A & -B \end{bmatrix} \cdot \mathbf{P}(t),$$

with $\mathbf{P}(t) = \left[P_{C_0}(t) \quad P_{C_1}(t) \quad P_{C_2}(t) \quad P_{C_3}(t) \quad P_{C_4}(t) \quad P_O(t)\right]^T$. The conductance G_M is then given by

$$G_M = N \cdot g_k \cdot [0 \quad 0 \quad 0 \quad 0 \quad 0 \quad 1] \cdot \mathbf{P}(t) = N \cdot g_k \cdot P_O \, .$$

Equation with rate constants:

$$
\mathbf{P}_{k+1} = \begin{bmatrix} 1-4\alpha_r & \beta_r & 0 & 0 & 0 & 0 \\ 4\alpha_r & 1-3\alpha_r-\beta_r & 2\beta_r & 0 & 0 & 0 \\ 0 & 3\alpha_r & 1-2\alpha_r-2\beta_r & 3\beta_r & 0 & 0 \\ 0 & 0 & 2\alpha_r & 1-\alpha_r-3\beta_r & 4\beta_r & 0 \\ 0 & 0 & 0 & \alpha_r & 1-4\beta_r-A_r & B_r \\ 0 & 0 & 0 & 0 & A_r & 1-B_r \end{bmatrix} \cdot \mathbf{P}_{\boldsymbol{k}} ,
$$

The voltage-dependent transition probabilities are given by $\alpha = a \cdot \exp^{\left(\frac{V_M}{m}\right)}$ and $\beta = c \cdot \exp^{\left(-\frac{V_M}{n}\right)}$, with V_M potential difference over the cell membrane, whereas the transition rates α_r are given in s^{-1} and $\alpha = \alpha_r \cdot \Delta t$, with Δt… sampling interval. Note that the effect of the capacitance C_P was neglected in the simulations, E_{VS} was set to zero.
